# Cardiac MR reliably identifies patients with clinically significant left ventricular noncompaction using a novel mass quantification technique

**DOI:** 10.1186/1532-429X-17-S1-P64

**Published:** 2015-02-03

**Authors:** Brian L Dubin, Iram T Azam, Daniel C Lee, Kameswari Maganti, James C Carr, Jeremy D Collins

**Affiliations:** 1 Radiology, Northwestern University, Chicago, IL, USA; 2 Cardiology, Northwestern University, Chicago, IL, USA

## Background

Left ventricular noncompaction (LVNC) is a diagnosis often made at imaging with uncertain prognostic value. Jenni echocardiogram and Petersen cardiac MR (CMR) criteria utilize a single long-axis view to quantify the maximal ratio of noncompacted to compacted myocardial thickness, but these have not been shown to correlate with clinical status or disease progression. Quantitation of the trabecular mass is now simple to perform using advanced post-processing tools. Prior studies demonstrated that elevated LV trabeculated mass helps differentiate patients with LVNC from healthy controls and patients with other non-ischemic cardiomyopathies, but distinguishing patients with morphologic LVNC (M-LVNC) from those with clinically significant LVNC (CS-LVNC) remains problematic. The purpose of this study was to evaluate the performance of trabecular mass quantification at CMR to differentiate patients with CS-LVNC and M-LVNC.

## Methods

Retrospective analysis of 36 consecutive patients with a CMR meeting Petersen's criteria and an available echocardiogram. Two independent observers quantified noncompacted and compacted myocardial mass at end-diastole on QMass 7.6 (Medis, Leiden, Netherlands) using bright blood short-axis cine images. The noncompacted myocardial mass index (NCMMI) was calculated by dividing trabecular volume by body surface area and multiplying by 1.05 g/mL, the density of myocardium. CS-LVNC was defined by a clinical history of heart failure, ventricular arrhythmia, or cardioembolic event. Two-tailed t-tests were applied to compare NCMMI between groups. Subgroup analysis was performed by excluding subjects with negative echocardiograms by Jenni criteria and an ejection fraction (EF)>55%. Receiver operating characteristic (ROC) analysis was used to estimate cutoff values, sensitivities, and specificities. Interobserver agreement was assessed using the intraclass correlation coefficient (ICC).

## Results

22 patients met criteria for CS-LVNC (50% male, 45۰ years-old) versus 14 patients with M-LVNC (57.1% male, 44۰7 years-old). Those with CS-LVNC exhibited significantly greater NCMMI compared to those with M-LVNC (60.8 vs 47.2 mL, p= 0.020). ROC analysis demonstrated fair discrimination between CS-LVNC and M-LVNC (AUC 0.74, p=0.018), which significantly improved after excluding 11 patients with EF≥55% (AUC 0.87, p=0.002) and even further after excluding an additional 10 patients with negative echocardiograms (AUC 0.96, p=0.003) yielding ideal cutoff values and test characteristics presented in Table 1. Interobserver agreement was excellent for NCMMI measurements (ICC=0.85, p<0.001). Ratios of noncompacted to compacted mass and global myocardial mass were not significantly different between patients with CS-LVNC and M-LVNC (P≥0.05).

**Table 1:** ROC Analysis of NCMMI for CS-LVNC vs. M-LVNC.

	All patients (n=36)	EF<55% (n=25)	EF<55% and negative echo (n=15)
**AUC**	0.74	0.87	0.96

**P-value**	0.018	0.002	0.003

**Ideal Cutoff Value (mL)**	54.0	53.5	54.8

**Sensitivity (%)**	72.7	100	100

**Specificity (%)**	71.4	70.0	83.3

**PPV (%)**	80.0	83.3	90.0

**NPV (%)**	62.5	100	100

## Conclusions

Trabecular mass quantification at CMR provides an accurate and reliable means of identifying patients with clinically significant LV noncompaction, particularly among patients with a depressed EF and negative echocardiogram.

## Funding

None.

**Figure 1:**
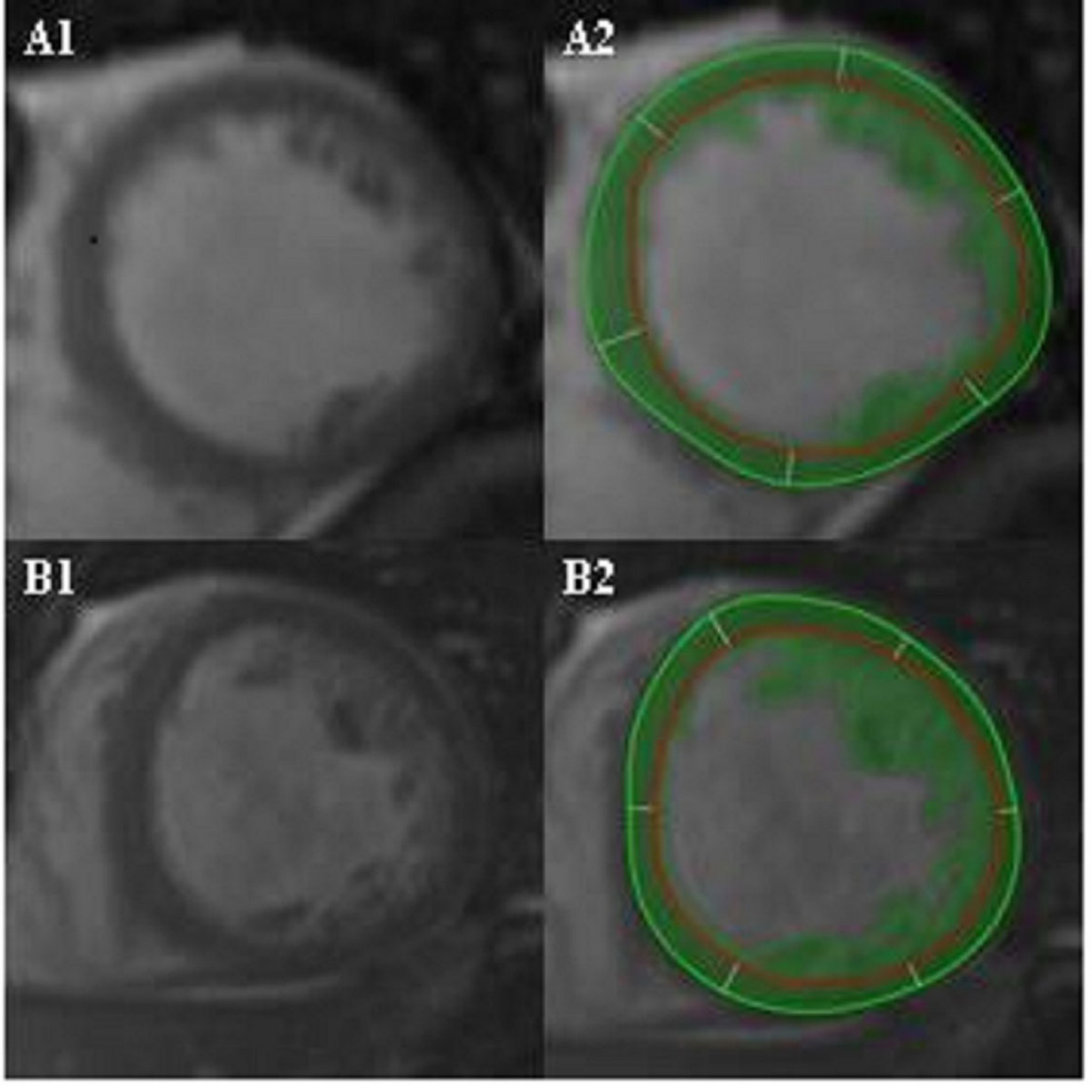
**Mid-chamber segments of two patients with LVNC by Petersen criteria.** Patient A has M-LVNC and NCMMI of 29.9 g/m^2^ vs. patient B with CS-LVNC and NCMMI of 102.6 g/m^2^. Images A2 and B2 depict epicardial contours (green circle), endocardial contours (red circle), and myocardial/trabeculated mass (green coloring).

